# Beyond the beats: exploring the link between blood pressure fluctuations with anxiety, depression, and sleep

**DOI:** 10.3389/fpsyt.2025.1595979

**Published:** 2025-07-31

**Authors:** Yuvthi Lutchman, Suzanne M. Cosh, Fiona Cossey, Deborah A. Turnbull, Rosannah Byrt, Cassandra Sundaraja, Rakesh Agarwal, Rajiv Mahajan, Christophe Tzourio, John F. Beltrame, Phillip J. Tully

**Affiliations:** ^1^ School of Psychology, The University of New England, Armidale, NSW, Australia; ^2^ School of Psychology, The University of Adelaide, Adelaide, SA, Australia; ^3^ Cardiac Surgery Research, Flinders Medical Centre, Adelaide, SA, Australia; ^4^ Adelaide Medical School, The University of Adelaide, Adelaide, SA, Australia; ^5^ Department of Cardiology, Lyell McEwin Hospital, Adelaide, SA, Australia; ^6^ University of Bordeaux, INSERM, Bordeaux Population Health Research Center, CHU Bordeaux, Bordeaux, France; ^7^ School of Medicine, The University of Adelaide, Adelaide, SA, Australia; ^8^ Department of Cardiology, The Queen Elizabeth Hospital, Adelaide, SA, Australia; ^9^ School of Psychology, Deakin University, Geelong, VIC, Australia

**Keywords:** ambulatory blood pressure monitoring, cardiovascular disease, cerebrovascular disease, arterial pressure, anxiety, depression, sleep

## Abstract

**Introduction:**

Blood pressure variability (BPV) is a marker of vascular and autonomic regulation, and emerging evidence links BPV with anxiety and depression. Limited research has explored central BPV, and secondly whether sleep modulates the association between BPV with anxiety and depression.

**Study design:**

Eighty-eight adults from clinical and community settings underwent 24-hour ambulatory blood pressure monitoring to assess central and brachial BPV, including augmentation index (AIx), mean arterial pressure (MAP), and pulse pressure (PP). Psychological symptoms were evaluated using the Generalized Anxiety Disorder Scale (GAD-7), Patient Health Questionnaire (PHQ-9), and Sleep Condition Indicator (SCI). Correlation and regression analyses were conducted, adjusting for age and gender. Mediation analyses explored the role of sleep disturbances in BPV-mood relationships.

**Results:**

Higher central BPV was associated with lower anxiety symptom severity, for somatic and cognitive symptoms (e.g., trouble relaxing: rs = -0.28, p < 0.01), whereas brachial BPV showed minimal associations. No significant relationships emerged between BPV and depressive symptoms after adjustment. AIx demonstrated significant negative associations with sleep-related issues, with stronger effects seen when adjusting for age and gender (β = -0.04, p < 0.01). Mediation analysis revealed sleep-related issues partially mediated the BPV-anxiety relationship.

**Discussion:**

Findings suggest that central BPV is more strongly associated with cognitive and somatic anxiety symptoms than depressive symptoms, with sleep disturbances potentially mediating this relationship. These results support BPV’s role in autonomic dysfunction, emphasizing the need for longitudinal research to clarify its role in mental health.

## 1 Introduction

Psychological distress is highly prevalent among individuals with cardiovascular disease (CVD), with anxiety and depressive symptoms occurring at significantly higher rates than in the general population (1). Anxiety symptoms, including excessive worry, restlessness, and heightened physiological arousal, and depressive symptoms, such as low mood, fatigue, and loss of interest, frequently coexist and are particularly common in heart failure (HF) patients, where one in five individuals experience clinically significant depressive symptoms and 13% meet criteria for an anxiety disorder (2, 3). These symptoms are strongly linked to disease progression and mortality, with greater symptom severity increasing the risk of major adverse cardiac events (4, 5). Beyond worsening cardiovascular outcomes, psychological distress also contributes to reduced quality of life, yet anxiety and depressive disorders often remain underdiagnosed and undertreated in CVD populations (6).

The relationship between cardiovascular and psychological health is complex, with growing evidence suggesting that blood pressure variability (BPV) may play a key role in this association. Unlike mean blood pressure, BPV reflects fluctuations over time, providing insights into vascular function, autonomic regulation, and cerebrovascular health (7). BPV is strongly linked to cardiovascular risk and influences key regulatory systems, including baroreflex sensitivity, autonomic nervous system (ANS) function, and cerebral blood flow (CBF) (8, 9). The ANS regulates involuntary physiological functions to maintain homeostasis, such as respiration, heart rate, body temperature, and digestive processes (10), all of which can be disrupted in individuals experiencing psychological distress (11). CBF is essential for brain health, ensuring adequate perfusion to mood-regulating regions including the prefrontal cortex, temporal lobes, and hippocampus (12). Disruptions in these systems may contribute to distinct symptom profiles, with ANS dysfunction potentially linked to somatic symptoms (e.g., dizziness, fatigue, breathlessness) and CBF dysregulation shown to be associated with cognitive symptoms (e.g., impaired concentration, rumination) (11, 12). These findings suggest that BPV may be a critical factor in understanding the interplay between CVD and mental health.

While BPV is known to impact autonomic and cerebrovascular stability, emerging evidence suggests that sleep disturbances may further modulate these effects. Sleep plays a crucial role in regulating BPV by maintaining autonomic balance, cardiovascular stability, and neurovascular function (13). Disruptions in blood pressure regulation during sleep reflect autonomic instability and may exacerbate BPV-related cardiovascular and psychological risks (14). Blunted nocturnal dipping and excessive morning BP surges—key features of circadian BP dysregulation—may further contribute to cardiovascular strain and autonomic instability, increasing the risk of stroke, myocardial infarction, and sudden cardiac death (15). Given these interactions, ambulatory blood pressure monitoring (ABPM), which captures both daytime and nighttime BP fluctuations, provides a more comprehensive perspective on BPV’s influence on cardiovascular and mental health (16).

Beyond its cardiovascular implications, sleep disturbances are strongly associated with anxiety and depressive symptoms, though their impact varies based on specific sleep dimensions (17, 18). Sleep-related patterns, including unstable circadian rhythms and irregular sleep duration, have been linked to increased BPV, autonomic dysfunction, and hypertension risk (14). Sleep-related daytime functioning issues, such as fatigue, excessive sleepiness, and cognitive impairment, are associated with greater anxiety sensitivity and depressive symptoms (17, 18). Notably, excessive daytime sleepiness appears more strongly linked to anxiety than depression, underscoring the importance of distinguishing between different types of sleep dysfunction when investigating BPV’s role in psychological distress (18). Given that sleep influences both autonomic and cerebrovascular regulation, exploring its mediating role in the BPV-mental health relationship could provide valuable insights into the physiological pathways underlying anxiety and depression.

Despite growing recognition of BPV’s role in anxiety, depression, and sleep quality, several methodological inconsistencies limit a clear understanding of these relationships. BPV measurement lacks standardization, with varying methods such as beat-to-beat (B2B), 24-hour ambulatory BPV, and visit-to-visit (V2V) BPV, leading to conflicting findings (19). Most research primarily examines brachial BPV, even though evidence suggests that central blood pressure variability (central BPV) which reflects pressure fluctuations in large arteries close to the heart and brain, may be a more accurate indicator of cerebrovascular function and autonomic regulation (20, 21). Central BPV reflects the pressure experienced by organs such as the brain and heart and is more closely linked to cerebrovascular integrity and arterial stiffness (22). Due to its proximity to central arteries, it may better capture autonomic dysregulation and wave reflection phenomena than brachial BPV, which is more susceptible to peripheral amplification and may underestimate central vascular load (23, 24). As such, central BPV may offer a more direct marker of physiological processes implicated in mood and sleep disturbances. Additionally, circadian BP fluctuations are rarely considered despite evidence linking daytime BPV to cognitive function and nocturnal BPV to brain morphology and total brain volume reduction in regions associated with mood regulation (25, 26). Current research rarely integrates circadian BP patterns with central BPV metrics, such as pulse pressure (PP), augmentation index (AIx), and mean arterial pressure (MAP), limiting understanding of BPV’s role in vascular health and psychological distress (27). Pulse pressure refers to the difference between systolic and diastolic blood pressure and is considered a marker of arterial stiffness and vascular load. Augmentation index (AIx) reflects the extent of wave reflection in the arteries and serves as an indirect measure of arterial stiffness and vascular aging. These central BP parameters, derived from pulse wave analysis, offer insights into vascular function that may be more sensitive to cerebral and autonomic influences than traditional brachial measures (28). While ABPM-derived measures serve as proxies for central BPV (24), their potential value in capturing BPV-related effects remains underexplored.

Additionally, while some studies analyze individual symptoms, such as hopelessness, others focus solely on total symptom scores, overlooking the importance of symptom dimensions (29). A multi-level approach that integrates individual symptoms, total scores, and cognitive and somatic dimensions is needed to capture BPV’s role in psychological distress comprehensively. Furthermore, previous studies vary in how they quantify BPV, using metrics such as standard deviation (SD), coefficient of variation (CV), and average real variability (ARV) (19). While SD is widely used, it primarily reflects dispersion around the mean and is sensitive to low sampling frequencies, potentially limiting its accuracy (30). In contrast, CV, which expresses SD as a ratio of mean BP, accounts for interindividual variability, making it more suitable for heterogeneous samples by emphasizing extreme fluctuations (30).

The present study aims to address these limitations by examining the associations between ambulatory BPV and symptoms of anxiety and depression. Specifically, it examines whether central and brachial BPV is associated with anxiety and depressive symptoms and whether cognitive and somatic symptom dimensions offer greater insight into these associations. Additionally, it investigates the role of sleep disturbances, including sleep patterns and daytime dysfunction, in relation to BPV and explores whether these symptoms mediate BPV’s effects on psychological distress. A dimensional approach to symptom assessment was adopted to capture nuanced associations between BPV and distinct somatic and cognitive domains of psychological distress, which are often masked in total score analyses. Additionally, using 24-hour ambulatory central and brachial BPV metrics allows for a more physiologically precise evaluation of cerebrovascular and autonomic contributions to mood and sleep disturbances, expanding on prior research limited to beat-to-beat measures. It was hypothesized that (1) central BPV would demonstrate stronger associations with anxiety symptoms than with depressive symptoms, reflecting its relevance to autonomic and cerebrovascular regulation; (2) these associations would be more pronounced in cognitive and somatic symptom dimensions than in global scores; and (3) sleep disturbances would mediate the relationship between BPV and anxiety and depressive symptoms, given sleep’s modulatory role in autonomic balance and cardiovascular regulation. By identifying central BPV as a potential physiological marker of psychological distress, this research may inform targeted screening and risk-stratification efforts in cardiovascular populations (31). Furthermore, understanding the mediating role of sleep may guide the development of integrated behavioural and physiological interventions that improve both mental and cardiovascular health outcomes (14, 17).

## 2 Methods

This study is an explorative, cross-sectional, quantitative observational study.

### 2.1 Participants

Participants with and without coronary artery disease (CAD), hypertension, or atrial fibrillation were recruited from three tertiary hospitals in metropolitan Adelaide, South Australia (The Queen Elizabeth Hospital, the Royal Adelaide Hospital, and the Lyell McEwin Hospital), medical centers based in Sydney, New South Wales, and the general Adelaide population. Eligibility included age 18 years or older, English language proficiency, and willingness to complete the 24-hour blood pressure monitoring. Participants were ineligible if they had a history of a recent transient ischemic attack (TIA), stroke, acute myocardial infarction (MI), or decompensated heart failure in the past six months, dementia or neurodegenerative conditions (e.g. Parkinson’s Disease), traumatic brain injury, or significant head trauma, a diagnosis of any life-limiting medical condition likely to be fatal within a year, alcohol or substance use disorders, psychosis or other severe psychiatric conditions requiring hospitalization, or a high risk of self-harm. The aim was to include a heterogeneous sample reflective of real-world cardiovascular risk profiles across clinical and non-clinical populations obtained from cardiology out-patient, primary care, and general populations.

Six of the 94 participants were excluded for having severe psychiatric conditions (*n* = 2) or being unable to wear the ABPM for 24 hours (*n* = 4). The final sample size was 88. Participation was voluntary, and those recruited from the general community were compensated with a Visa gift card of $50.00. This study was approved by the Human Research Ethics Committees of Central Adelaide Local Health Network (CALHN No 11733), The University of Adelaide (HREC/15/TQEH4), and the University of New England (Approval No HRE23-004).

### 2.2 Procedure

**Figure 1:**
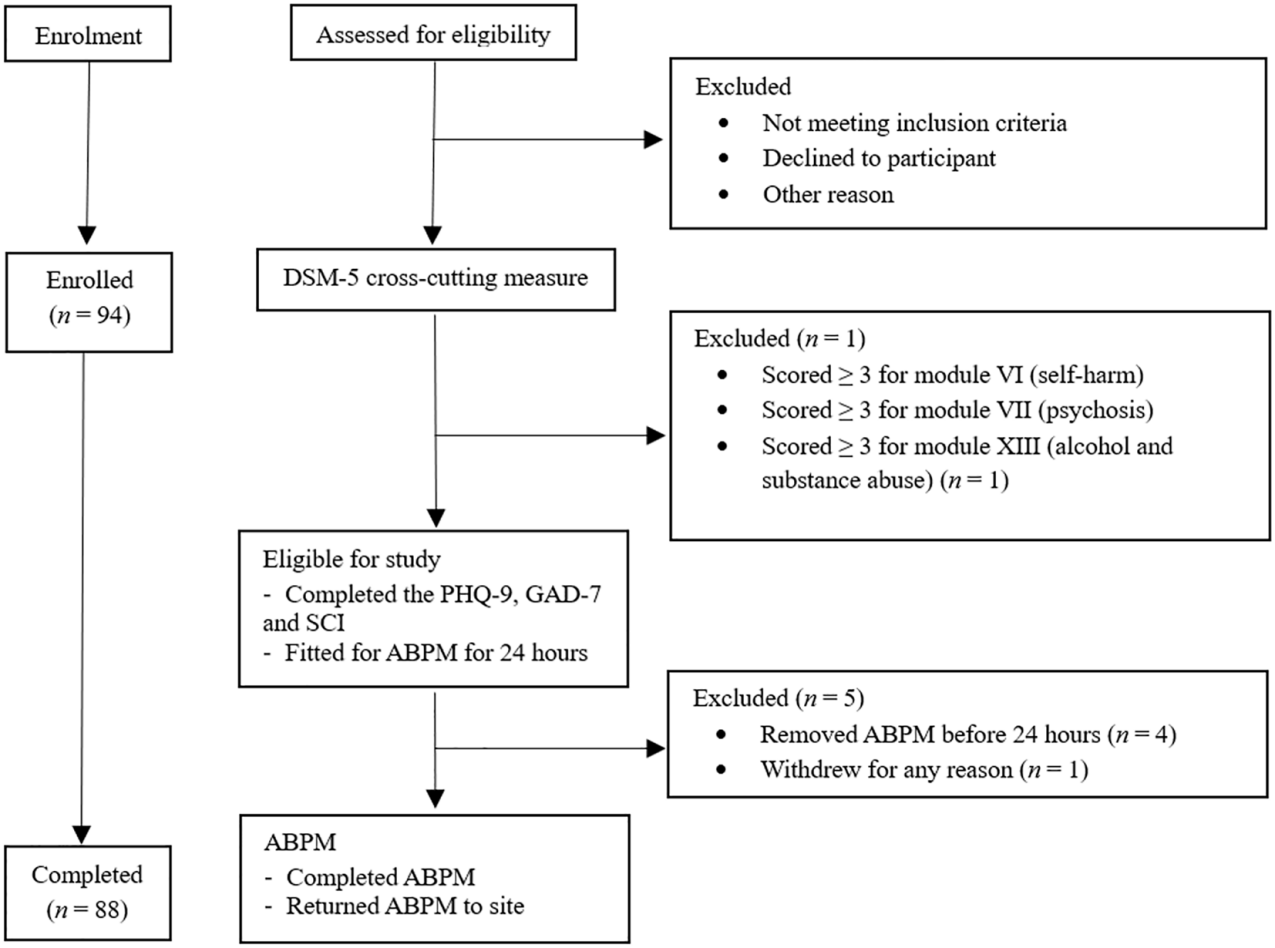
Study Procedure. Figure showing the flow of participants through the study, the study procedural timeline and reasons for exclusion. *ABPM* Ambulatory blood pressure monitor, *PhQ-9* Patient Health Questionnaire- 9, *GAD-7* Generalised Anxiety Disorder Assessment-7, *SCI* Sleep Quality Assessment, *DSM-5* Diagnostic Statistical Manual of Mental Disorders – fifth edition.

All participants received an information sheet and completed eligibility screening via phone or electronically. Written consent and demographic information were obtained, followed by an eligibility screener, the DSM-5 Self-Rated Level 1 Cross-Cutting Symptom Measure – Adult (DSM-5 XC), used to screen for alcohol or substance use disorders, psychosis, or other psychiatric conditions in individuals as part of the exclusion criteria process. The 23-item questionnaire assessed 13 psychiatric domains, including depression, anger, mania, anxiety, somatic symptoms, suicidal ideation, psychosis, sleep problems, memory, repetitive thoughts and behaviors, dissociation, personality functioning, and substance use (32). The DSM-5 XC is clinically validated in screening for psychiatric disorders and has good test-retest reliability in the DSM-5 Field Trials conducted in adult clinical samples internationally (32). Participants were excluded if they scored ≥ 3 for modules of self-harm, psychosis, or alcohol and drug abuse and were referred to their general practitioner as part of duty of care. Eligible participants then proceeded to complete measures of cognitive function (not reported further), depression, anxiety, and blood pressure (See **Figure 1**).

### 2.3 Blood pressure variability

#### 2.3.1 Ambulatory blood pressure monitoring

Standardized 24-hour ABPM was performed using a calibrated and validated device (SunTech^®^ Oscar 2 BP monitor). The Oscar 2 (SunTech^®^ BP Monitor) is a non-invasive oscillometric device validated to record blood pressure with a standard error of +/- 5 mmHg. It has been clinically validated, having been tested to meet the accuracy and performance requirements of the Association for the Advancement of Medical Instrumentation and the International Organization of Standardization, as it utilizes motion-tolerant technology to deliver fewer failed readings compared to other ABPM monitors. The monitor was programmed for automated recordings every 20 minutes during awake periods and every 30 minutes during asleep periods, typically yielding up to 45 awake and 18 asleep recordings. Participants were fitted with an appropriately sized BP cuff on their left upper arm, and a waist belt was provided to hold the ABPM. Following standardized 24-hour ABPM protocol, all participants were instructed to limit certain physical activities, avoid wetting the device, maintain sleep and wake times, and wear the ABPM continuously without breaks for 24 hours in their home environment. In cases of discomfort, they were informed how to remove the device early and withdraw from the study. Follow-up occurred 24 hours later to debrief, remove, and collect the ABPM.

#### 2.3.2 Blood pressure metrics

Using pulse wave analysis (PWA) and a mathematical model, the Suntech Oscar 2 ABPM calculates central BPV proxies that mimic invasive catheter readings. The monitor recorded a range of central and brachial BP phenotypes, including central and brachial SBP and DBP, MAP, PP, and central AP and AIx for arterial stiffness. In addition to overall (24-hour) BP, ambulatory BP data was subdivided into awake and asleep data. All BP data was analyzed through the AccuWin Pro 4 ABPM software (SunTech Medical). BP mean and CV values were automatically recorded with the AccuWin Pro 4 ABPM software (SunTech Medical).

### 2.4 Severity of anxiety symptoms

The Generalized Anxiety Disorder scale (GAD-7) (33), is a 7-item brief questionnaire that be used to measure symptoms of anxiety. Items are based on the diagnostic criteria for Generalized Anxiety Disorder defined in the DSM-5 (32). This includes symptoms of nervousness, uncontrollable and excessive worry, difficulty relaxing, restlessness, irritability, and feeling afraid. Each item in the GAD-7 is scaled from “0” (not at all) to “3” (nearly every day), with sum scores representing severity. The GAD-7 scale can be effectively divided into cognitive-emotional and somatic components. Cognitive questions focus on worry and mental tension, while somatic questions address physical symptoms like restlessness and irritability (34). The scale has been found to have good internal consistency (α = 0.89) (33).

### 2.5 Severity of depression symptoms

As per the screening recommendations of the American Heart Association (35), the Patient Health Questionnaire (PHQ-9) (36), was used to quantify depressive symptoms. The PHQ-9 is a 9-item self-administered scale that measures symptoms requisite for the diagnosis of depression per the DSM-5 (32). Symptoms measured include diminished interest or pleasure, hopelessness, sleep difficulties and fatigue, changes in appetite or weight, feeling like a failure, concentration difficulties, psychomotor agitation, and suicidal ideations. Like the GAD-7, each item is scaled from “0” (not at all) to “3” (nearly every day), with sum scores representing severity. Similar to the GAD-7, the PHQ-9 has a bi-dimensional structure, with somatic (sleep problems, fatigue, appetite problems, and psychomotor difficulties) and cognitive-affective (lack of interest, depressed mood, negative feelings about self and concentration) dimensions (37). The PHQ-9 is a short and simple measure with good psychometric properties, including construct and criterion validity and internal consistency (α of 0.89) (36). Both the GAD-7 and PHQ-9 have been used in Australian cardiovascular populations including cardiac surgery (38), heart failure (39), and general CVD populations.

### 2.6 Sleep disturbance

The Sleep Condition Indicator (SCI) is an eight-item self-report scale developed to screen for insomnia disorder in line with DSM-5 criteria (40) as well as sleep quality and insomnia symptoms in research and clinical settings. This instrument assesses key areas related to sleep difficulties, including problems with initiating and maintaining sleep, subjective sleep quality, impact on daytime functioning and performance, duration and frequency of sleep disturbances, and distress caused by poor sleep. Each item is rated on a five-point scale from 0 to 4 (40). The SCI demonstrates excellent psychometric properties, with robust internal consistency (α ≥ 0.86) and convergent validity with the Pittsburgh Sleep Quality Index and the Insomnia Severity Index. The SCI was reverse scored to align with the directionality of the GAD-7 and PHQ-9 so that higher SCI scores indicated greater severity of sleep disturbance and poorer functioning (41).

### 2.7 Statistical analysis

Statistical analyses were performed using IBM SPSS Statistics (Version 29; IBM Corp, 2023). Data normality was assessed using the Shapiro-Wilk test. Extreme BP readings were screened, with exclusion thresholds set at systolic >240 mmHg or <50 mmHg and diastolic >140 mmHg or <40 mmHg; no data met these exclusion criteria. Spearman’s correlations were conducted to assess the relationships between central and brachial BPV and individual symptom items and total scores from the GAD-7, PHQ-9 and SCI questionnaires across ambulatory, awake, and asleep periods. Multiple linear regressions were employed to examine the independent relationships between BPV metrics and these selected factors across all three time periods. Model 1 assessed unadjusted associations, while Model 2 accounted for potential confounders by adjusting for age and gender. Outliers were identified using standard residuals (≥ 3); however, none were detected. Subsequently, mediation analyses were conducted to investigate the mediating role of sleep in the relationship between BPV and mood outcomes. Given the cross-sectional nature of the data, mediation models were used for exploratory purposes to examine potential indirect associations, without inferring causal or temporal direction. Using the PROCESS macro of SPSS, bootstrapping with 10,000 resamples was applied to estimate confidence intervals for indirect effects. The regression model (unadjusted or adjusted) with the most consistent associations was chosen for the mediation analysis, ensuring a robust exploration of BPV and mood outcomes. As an exploratory study, no corrections for multiple comparisons were applied (42, 43).

## 3 Results

### 3.1 Participant characteristics

The final sample comprised 88 participants (females *n* = 34, 39%) across South Australia (*n* = 69) and Sydney (*n* = 19). The median age was 57.6 years old (range 23 – 86). Participants’ body mass index (BMI) ranged between healthy and obese. More than half of the participants were employed or self-employed (56%). Approximately 67% of participants were non-smokers, and most had a university degree. The most prevalent cardiovascular disease among the population was hypertension (32%), followed by coronary artery disease (21%) and atrial fibrillation (11%) (see **Table 1**), supporting generalizability to mid-aged adults with and without cardiovascular risk.

**Table 1:** Demographic Characteristics of Participants (n = 88).

Demographic Characteristics	
Gender, n (%)	
Females	34 (39)
Males	54 (61)
Age in years, Median (range)	57.6 (23 – 86)
Body mass index, Median (range)	28.1 (22.7 – 37.5)
Education, n (%)	
Left formal education ≤18 years old	30 (34)
Undergraduate degree	33 (38)
Postgraduate degree	25 (28)
Employment Level, n (%)	
Employed or self-employed	49 (56)
Unemployed or retired	39 (44)
Smoking Status, n (%)	
Never	59 (67)
Former	25 (28)
Current	4 (5)
Cardiovascular health, n (%)	
Hypertension	28 (32)
Coronary artery disease	18 (21)
Atrial fibrillation	10 (11)
Myocardial infarction	15 (17)
Chronic Pain, n (%)	7 (8)

Caption: Data shown as N (%) unless otherwise stated.

### 3.2 Associations of individual symptom items and total scores

#### 3.2.1 Anxiety symptoms

**Figure 2:**
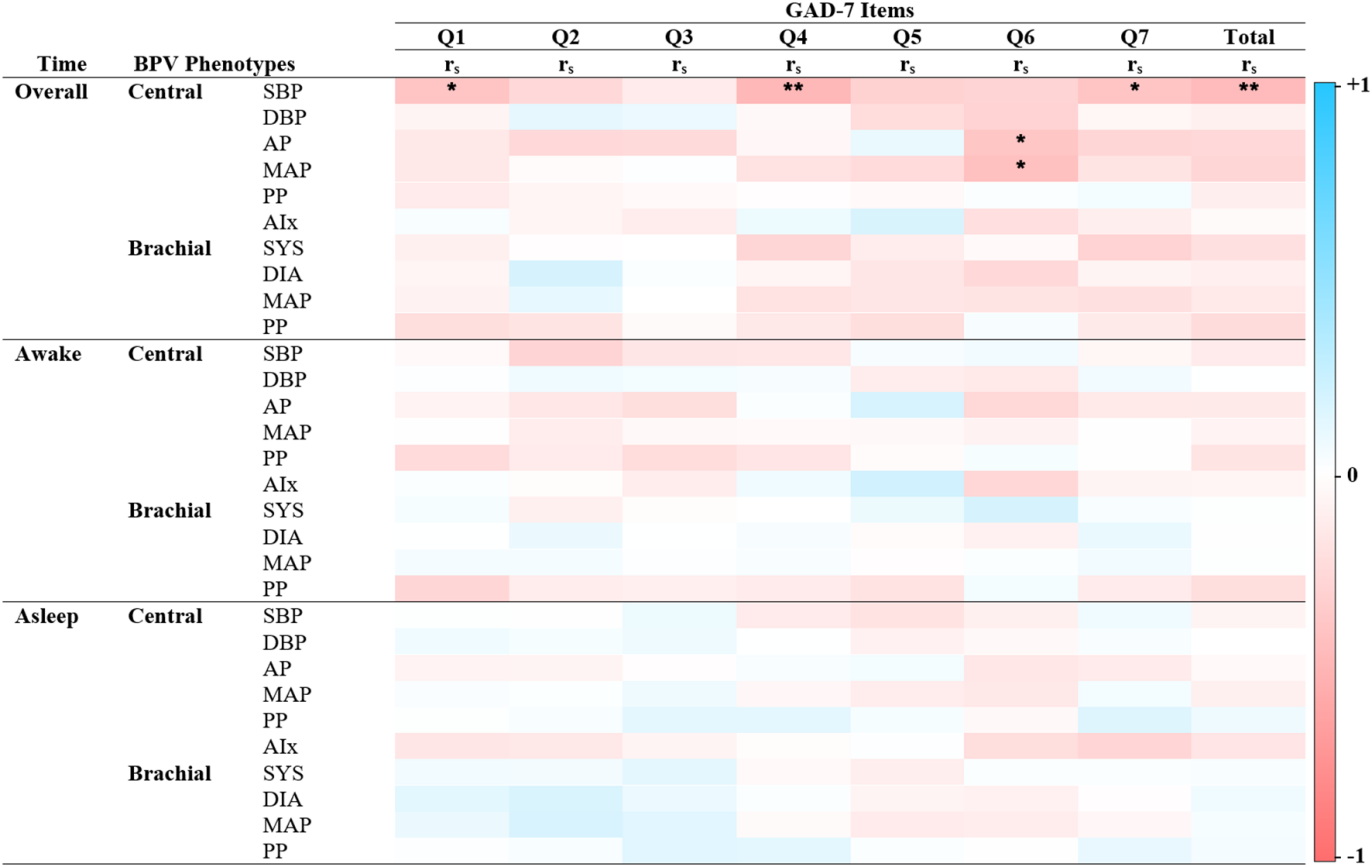
Spearman’s correlations of BPV and anxiety scores. Heatmap of the pairwise Spearman’s rho correlations between blood pressure variability (BPV) phenotypes and individual items from the Generalized Anxiety Disorder scale (GAD-7), stratified by 24-hour period (Overall, Awake, Asleep). Darker shades of red indicate strong positive correlations, while darker shades of blue indicate strong negative correlations. The color scale ranges from -1 (perfect negative correlation) to 1 (perfect positive correlation), with 0 indicating no correlation. GAD – 7 Item 1: Nervousness, Item 2: Uncontrollable worry, Item 3: Excessive worry, Item 4: Difficulty relaxing, Item 5: Restlessness, Item 6: Irritability, Item 7: Feeling afraid. Alx Augmentation Index of Arterial Stiffness, Augmentation Pressure, DBP Diastolic Pressure, MAP Mean Arterial Pressure, PP Pulse Pressure, rs Spearman’s rho correlation coefficient, SBP Systolic Pressure. *P < 0.05 ** P< 0.01.

**Figure 3:**
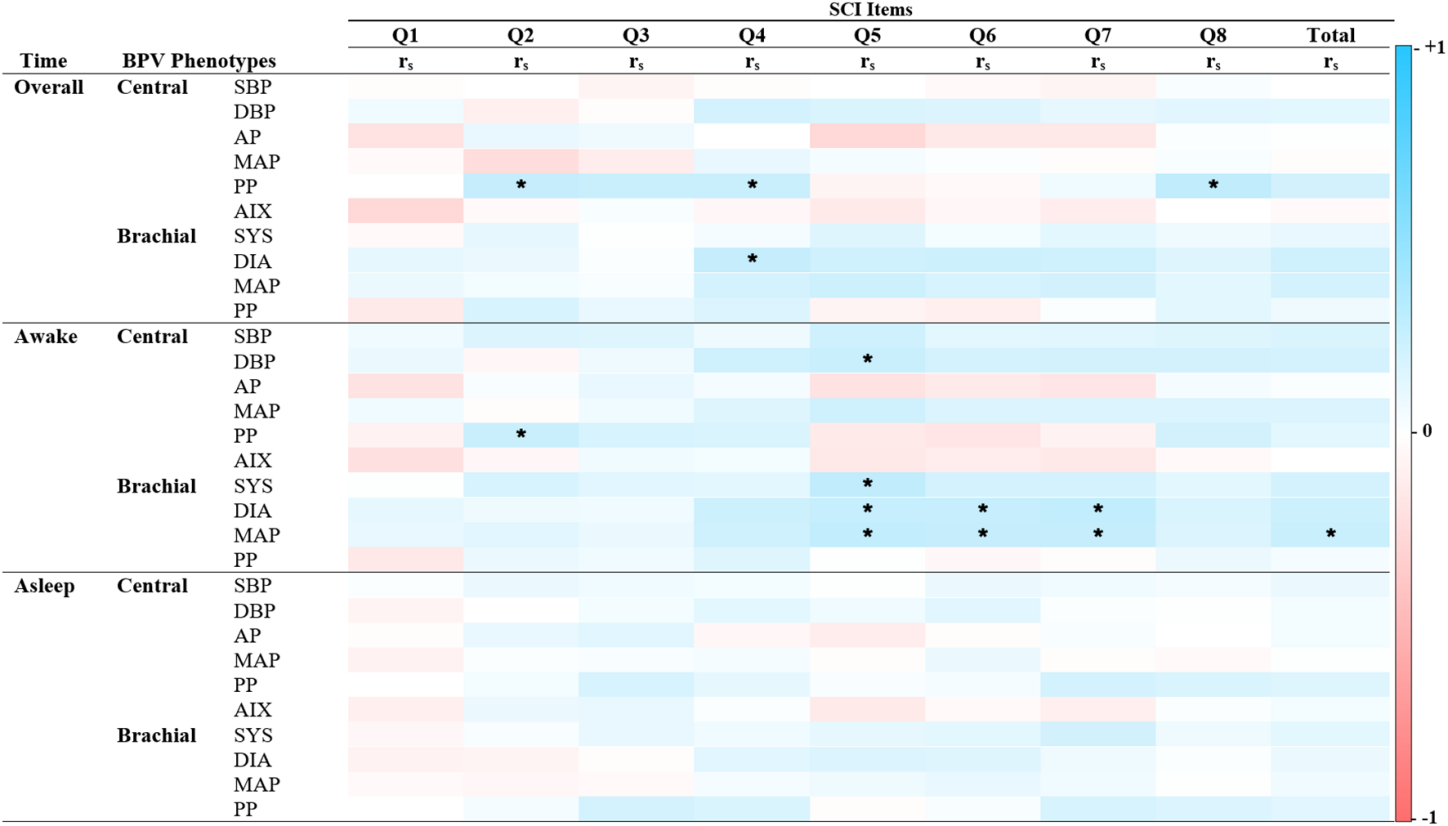
Spearman’s correlations of BPV and depression scores. Heatmap of the pairwise Spearman’s rho correlations between blood pressure variability (BPV) phenotypes and individual items from the Patient Health Questionnaire (PHQ-9), stratified by time period (Overall, Awake, Asleep). Darker shades of red indicate strong positive correlations, while darker shades of blue indicate strong negative correlations. The color scale ranges from -1 (perfect negative correlation) to 1 (perfect positive correlation), with 0 indicating no correlation. PHQ-9 Item 1: Diminished interest, Item 2: Hopelessness, Item 3: Sleep difficulties, Item 4: Fatigue, Item 5: Changes in appetite or weight, Item 6: Feeling like a failure, Item 7: Concentration difficulties, Item 8: Psychomotor agitation, Item 9: Suicidal ideations. Alx Augmentation Index of Arterial Stiffness, AP Augmentation Pressure, DBP Diastolic Pressure, MAP Mean Arterial Pressure, PP Pulse Pressure, r_s_ Spearman’s rho correlation coefficient, SBP Systolic Pressure. *P < 0.05.

**Figure 4:**
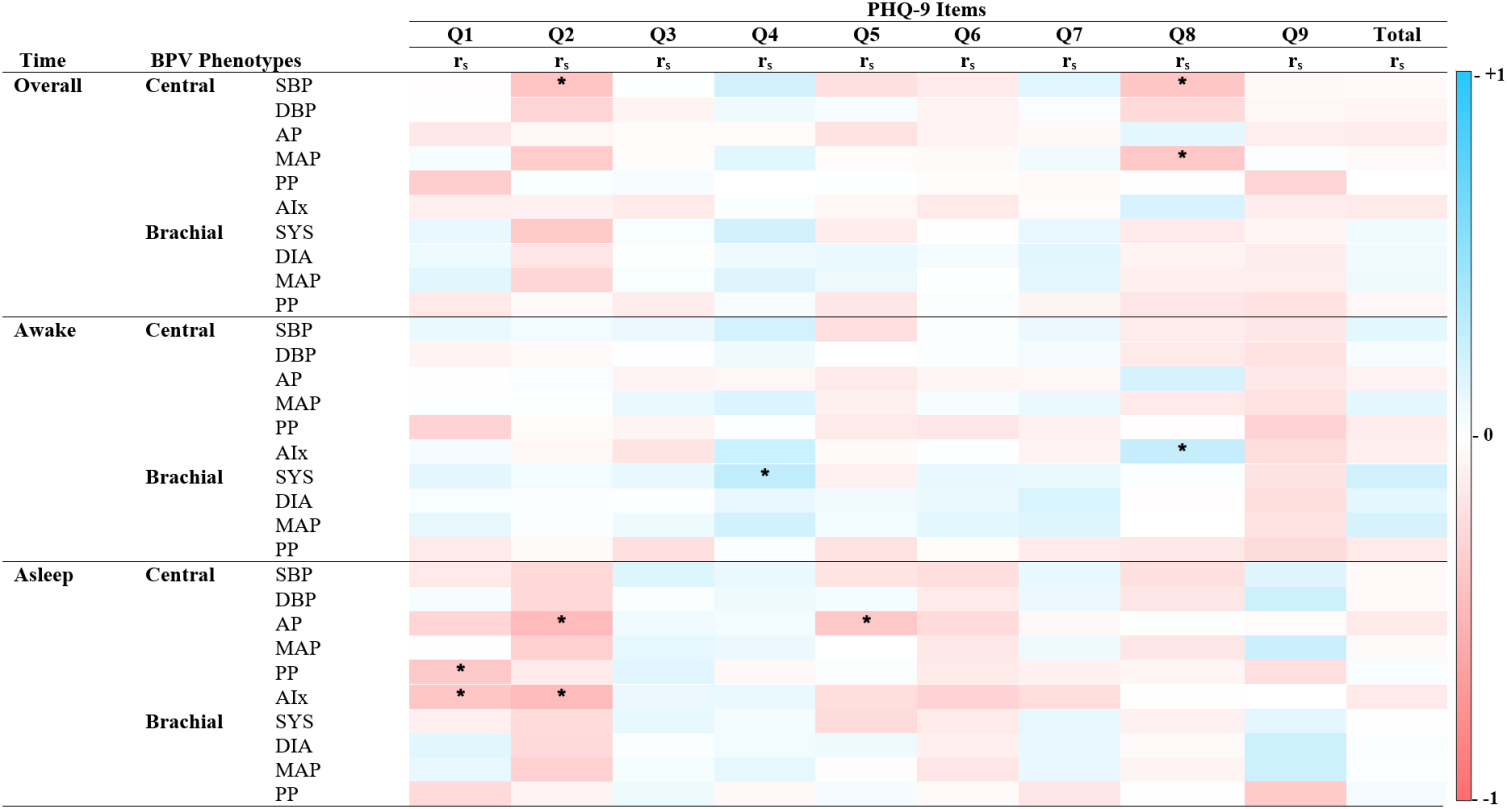
Spearman’s correlations of BPV and reversed scored sleep quality. Heatmap of the pairwise Spearman’s rho correlations between blood pressure variability (BPV) phenotypes and items from the Sleep Condition Indicator (SCI), stratified by time period (Overall, Awake, Asleep). Darker shades of red indicate strong positive correlations, while darker shades of blue indicate strong negative correlations. The color scale ranges from -1 (perfect negative correlation) to 1 (perfect positive correlation), with 0 indicating no correlation. SCI Item 1: Initiating sleep, Item 2: Length of sleep interruptions. Item 3: Frequency of difficulties. Item 4: Self-rated quality. Item 5: Impact on mood, energy or relationships. Item 6: Impact on concentration or productivity. Item 7: Distress. Item 8: Length of sleep problems. Alx Augmentation Index of Arterial Stiffness, AP Augmentation Pressure, DBP Diastolic Pressure, MAP Mean Arterial Pressure, PP Pulse Pressure, rs Spearman’s rho correlation coefficient, SBP Systolic Pressure. *P < 0.05.

Significant correlations were primarily observed between central BPV measures and anxiety outcomes during the overall and awake periods (**Figures 2**–**4**). Central systolic BPV showed negative associations with somatic symptom “Trouble relaxing” (rs = -0.28, p < 0.01) and cognitive symptom items “Feeling nervous, anxious or on edge” and “Feeling afraid” (rs = -0.21, p < 0.05; rs = -0.22, p < 0.05 respectively). Total GAD-7 score was also negatively associated with central SBP (rs = -0.27, p<0.01). Other central BPV metrics (AP and MAP) correlated with “Easily annoyed”. Overall, BPV-anxiety correlations were small in magnitude. No associations were observed between GAD-7 items and brachial BPV or during the sleep period (See **Figure 2**).

#### 3.2.2 Depressive symptoms

Significant correlations between BPV metrics and PHQ-9 items revealed distinct time- and factor-specific patterns (See **Figure 2**; **Supplementary Table 1**). During sleep, central BPV metrics (AP, PP, and Alx) negatively correlated with cognitive symptom items, including “Little interest or pleasure” and “Feeling down, depressed, or hopeless” (r_s_ = -0.22 to -0.26, p < 0.05). Across the full period, central systolic BPV was negatively associated with “Feeling down, depressed, or hopeless” and “Moving or speaking slowly” (rs = -0.23, p<0.05; rs = -0.22, p < 0.05 respectively). During the awake period, brachial systolic BPV was positively associated with somatic symptoms, particularly “Feeling tired or having little energy” (rs = 0.24, p < 0.05). Compared to central BPV, brachial BPV showed fewer significant correlations, mirroring patterns observed in the GAD-7 scale. No significant associations were found with total PHQ-9 score, highlighting the importance of symptom-specific level of analyses.

#### 3.2.3 Sleep quality symptoms

Unlike anxiety and depression, BPV correlations with sleep quality were predominantly positive (See **Figure 4**). Significant associations emerged during the 24-hour and awake periods, but none were found during sleep. Central PP positively correlated with reversed SCI sleep pattern items, including “Length of sleep interruptions” (rs = 0.23, p < 0.05), “Self-rated sleep quality” (rs = 0.21, p < 0.05), and “Length of sleep problems” (rs = 0.25, p < 0.05). Brachial BPV showed more frequent associations than central BPV, with systolic, diastolic, and MAP positively linked to “Impact on mood, energy, or relationships,” “Impact on concentration and productivity,” and “Length of distress”. Total SCI score was associated only with brachial MAP during the awake period (rs = 0.21, p < 0.05). These findings underscore time- and factor-specific BPV-sleep relationships and inform the subsequent regression analysis.

### 3.3 Regression analysis of symptoms

#### 3.3.1 Anxiety and depressive symptoms

Regression analysis identified significant associations between central BPV and GAD-7 total scores, particularly in the overall period (**Table 2**). Central SBPV and MAP negatively correlated with somatic anxiety symptoms (Central SBPV Model 1: β = -0.16, p = 0.01; Model 2: β = -0.14, p = 0.03). AIx was consistently linked to lower cognitive, somatic, and total anxiety scores across awake and asleep periods. These findings were absent in correlation analyses, emphasizing the importance of adjusting for demographic factors. In contrast, no significant relationships were found between BPV metrics and PHQ-9 symptoms (see **Table 3**; **Supplementary Table 2**), consequently leading to PHQ-9 symptoms being excluded from mediation analysis.

**Table 2:** Linear regressions of BPV and GAD-7 symptoms.

			Cognitive Symptoms		Somatic Symptoms		Total Score	
			Model 1	Model 2	Model 1	Model 2	Model 1	Model 2
** Time**	** BPV Phenotypes**		β	β	β	β	β	β
**Overall**	**Central**	SBP	-0.14	-0.09	-0.16*	-0.14*	-0.30*	-0.22
		DBP	0.02	0.00	-0.05	-0.06	-0.03	-0.06
		AP	-0.02	-0.03*	-0.01	-0.02*	-0.03	-0.05**
		MAP	-0.04	-0.05	-0.11*	-0.12*	-0.14	-0.17
		PP	-0.01	0.03	0.00	0.02	-0.01	0.05
		AIx	-0.01	-0.03*	0.00	-0.02	-0.02	-0.05*
	**Brachial**	SYS	-0.04	0.00	-0.08	-0.07	-0.12	-0.06
		DIA	0.01	0.00	-0.03	-0.03	-0.02	-0.03
		MAP	0.00	0.00	-0.05	-0.05	-0.05	-0.06
		PP	0.00	0.04	-0.01	0.01	0.00	0.05
**Awake**	**Central**	SBP	-0.08	-0.02	0.00	0.07	-0.08	0.05
		DBP	0.00	-0.01	0.00	0.00	0.00	0.00
		AP	-0.01	-0.02	0.00	-0.01	-0.02	-0.04
		MAP	0.00	0.01	0.00	0.03	0.00	0.04
		PP	-0.04	-0.01	0.00	0.01	-0.04	0.00
		AIx	-0.01	-0.02*	0.00	-0.02*	-0.01	-0.04*
	**Brachial**	SYS	0.02	0.06	0.06	0.10	0.08	0.16
		DIA	0.02	0.02	0.01	0.03	0.03	0.05
		MAP	0.00	0.00	-0.05	-0.05	-0.05	-0.06
		PP	-0.01	0.01	-0.01	0.00	-0.02	0.00
**Asleep**	**Central**	SBP	-0.01	0.02	-0.04	-0.05	-0.05	-0.04
		DBP	0.01	0.01	-0.02	-0.04	-0.01	-0.03
		AP	-0.01	-0.02	0.00	-0.01	-0.01	-0.03
		MAP	0.00	0.00	-0.02	-0.04	-0.02	-0.04
		PP	0.02	0.03	0.01	0.01	0.03	0.04
		AIx	-0.02	-0.03*	-0.01	-0.02*	-0.03	-0.04*
	**Brachial**	SYS	0.00	0.01	-0.01	-0.03	-0.01	-0.01
		DIA	0.03	0.02	-0.01	-0.04	0.02	-0.02
		MAP	0.01	0.00	-0.01	-0.02	0.00	-0.02
		PP	0.03	0.05	0.02	0.01	0.04	0.06

Caption: N = 88. Alx, Augmentation Index of Arterial Stiffness; AP, Augmentation Pressure; β, Regression Coefficient; BPV, Blood Pressure Variability calculated using the coefficient of variation; DBP, Diastolic Blood Pressure; GAD-7, Generalized Anxiety Disorder-7; MAP, Mean Arterial Pressure; PP, Pulse Pressure; SBP, Systolic Blood Pressure;

Model 1: Unadjusted, Model 2: Adjusted for Age and Gender.

Bolded values denoted *P <0.05 ** P< 0.01.

**Table 3:** Linear regressions of BPV and PHQ-9 symptoms.

			Cognitive Symptoms		Somatic Symptoms		Total Score	
			Model 1	Model 2	Model 1	Model 2	Model 1	Model 2
** Time**	** BPV Phenotypes**		β	β	β	β	β	β
**Overall**	**Central**	SBP	-0.06	-0.03	-0.07	-0.04	-0.13	-0.07
		DBP	-0.04	-0.05	-0.05	-0.06	-0.09	-0.12
		AP	-0.01	-0.01	0.00	-0.01	-0.01	-0.02
		MAP	-0.03	-0.03	-0.07	-0.07	-0.10	-0.10
		PP	-0.03	-0.01	0.00	0.02	-0.03	0.01
		AIx	-0.01	-0.01	0.00	-0.01	-0.02	-0.03
	**Brachial**	SYS	-0.03	-0.01	-0.05	-0.02	-0.08	-0.03
		DIA	0.01	0.00	-0.01	-0.02	0.00	-0.01
		MAP	0.00	-0.01	-0.02	-0.02	-0.02	-0.03
		PP	-0.02	0.00	-0.04	-0.01	-0.06	-0.01
**Awake**	**Central**	SBP	0.06	0.07	0.04	0.08	0.09	0.15
		DBP	-0.02	-0.03	-0.02	-0.03	-0.04	-0.05
		AP	-0.01	0.00	-0.01	-0.01	-0.01	-0.01
		MAP	0.02	0.01	0.00	0.00	0.02	0.01
		PP	-0.03	-0.02	-0.01	0.01	-0.04	-0.01
		AIx	-0.01	-0.01	-0.01	-0.01	-0.01	-0.02
	**Brachial**	SYS	0.05	0.06	0.06	0.08	0.11	0.14
		DIA	0.02	0.01	0.02	0.01	0.04	0.02
		MAP	0.00	-0.01	-0.02	-0.02	-0.02	-0.03
		PP	-0.02	-0.01	-0.05	-0.04	-0.07	-0.05
**Asleep**	**Central**	SBP	-0.03	0.00	0.02	0.04	-0.01	0.04
		DBP	-0.01	0.00	0.00	0.00	-0.01	0.00
		AP	-0.01	-0.01	0.00	-0.01	-0.01	-0.02
		MAP	-0.01	0.00	0.01	0.01	0.00	0.01
		PP	-0.03	-0.02	0.02	0.03	-0.01	0.02
		AIx	-0.01	-0.02	0.00	0.00	-0.01	-0.02
	**Brachial**	SYS	-0.03	-0.01	0.00	0.02	-0.03	0.01
		DIA	0.01	0.01	0.00	-0.01	0.01	0.00
		MAP	0.00	0.00	-0.01	-0.01	-0.01	-0.01
		PP	-0.02	-0.01	0.02	0.04	0.00	0.03

Caption: N = 88. Alx, Augmentation Index of Arterial Stiffness; AP, Augmentation Pressure; β, Regression Coefficient; BPV, Blood Pressure Variability calculated using the coefficient of variation; DBP, Diastolic Blood Pressure; MAP, Mean Arterial Pressure; PHQ-9, Patient Health Questionnaire-9; PP, Pulse Pressure; SBP, Systolic Blood Pressure;

Model 1: Unadjusted, Model 2: Adjusted for Age and Gender.

#### 3.3.2 Sleep quality symptoms

BPV-sleep associations varied by sleep factor and time period (**Table 4**). Central PP was the strongest predictor of sleep patterns, showing positive associations in the overall period (Model 1: β = 0.34, p = 0.04; Model 2: β = 0.37, p = 0.03) and with total SCI scores in Model 2 (β = 0.41, p < 0.05). Brachial PP, rather than central PP, correlated with poor sleep patterns and higher SCI scores during sleep (β = 0.22–0.29, p = 0.04). For sleep-related issues, AIx and central AP showed negative associations during the overall and awake periods (β = -0.04 to -0.03, p < 0.01), but were not significant during sleep. Conversely, brachial BPV metrics (diastolic BPV, MAP) were positively linked to sleep-related issues in the overall and awake periods. Brachial systolic BPV was the only metric significantly associated with the sleep period (β = 0.12, p < 0.05). These findings highlight distinct roles of central and brachial BPV in sleep dysfunction, supporting their inclusion in mediation analysis.

**Table 4:** Linear regressions of BPV and SCI symptoms.

			Sleep Patterns		Sleep-Related Functioning Issues		Total SCI	
			Model 1	Model 2	Model 1	Model 2	Model 1	Model 2
** Time**	** BPV Phenotypes**		β	β	β	β	β	β
**Overall**	**Central**	SBP	0.08	0.09	-0.02	0.05	0.06	0.13
		DBP	0.10	0.10	0.09	0.07	0.19	0.17
		AP	0.02	0.03	-0.01	-0.03*	0.00	0.00
		MAP	-0.02	-0.02	0.02	0.01	0.00	0.00
		PP	0.34*	0.37*	0.00	0.04	0.34	0.41*
		AIx	0.00	0.01	-0.02	-0.04**	-0.02	-0.02
	**Brachial**	SYS	0.18	0.20	0.07	0.12	0.26	0.33
		DIA	0.18	0.17	0.12**	0.11*	0.30*	0.28
		MAP	0.22	0.22	0.15*	0.14*	0.37	0.35
		PP	0.23	0.26	0.01	0.05	0.24	0.31
**Awake**	**Central**	SBP	0.28	0.27	0.12	0.20*	0.40	0.47
		DBP	0.04	0.04	0.03	0.03	0.08	0.06
		AP	0.01	0.02	-0.02	-0.03*	-0.01	0.00
		MAP	0.15	0.12	0.14	0.15*	0.29	0.27
		PP	0.25	0.26	-0.05	-0.02	0.20	0.24
		AIx	0.00	0.00	-0.02	-0.03**	-0.02	-0.03
	**Brachial**	SYS	0.30	0.28	0.20*	0.25*	0.50	0.53
		DIA	0.19	0.17	0.13**	0.13*	0.32*	0.31*
		MAP	0.22	0.22	0.15*	0.14*	0.37	0.35
		PP	0.14	0.15	0.01	0.02	0.15	0.17
**Asleep**	**Central**	SBP	0.13	0.17	0.06	0.09	0.18	0.26
		DBP	0.01	0.03	0.03	0.02	0.04	0.05
		AP	0.02	0.03	0.00	-0.01	0.02	0.03
		MAP	0.01	0.04	0.02	0.02	0.04	0.06
		PP	0.17	0.20	0.05	0.07	0.22	0.27
		AIx	0.01	0.02	-0.01	-0.01	0.00	0.00
	**Brachial**	SYS	0.14	0.18	0.09	0.12*	0.23	0.30
		DIA	0.05	0.07	0.06	0.05	0.11	0.11
		MAP	-0.02	-0.01	-0.01	-0.01	-0.02	-0.02
		PP	0.18	0.22*	0.04	0.06	0.22	0.29*

Caption; N = 88. Alx, Augmentation Index of Arterial Stiffness; AP, Augmentation Pressure; β, Regression Coefficient; BPV, Blood Pressure Variability calculated using the coefficient of variation; DBP, Diastolic Blood Pressure; MAP, Mean Arterial Pressure; PP, Pulse Pressure; SBP, Systolic Blood Pressure; SCI, Sleep Condition Indicator;

Model 1: Unadjusted, Model 2: Adjusted for Age and Gender.

Bolded values denoted *P < 0.05 ** P< 0.01.

### 3.4 Mediation analysis of sleep quality

**Figure 5:**
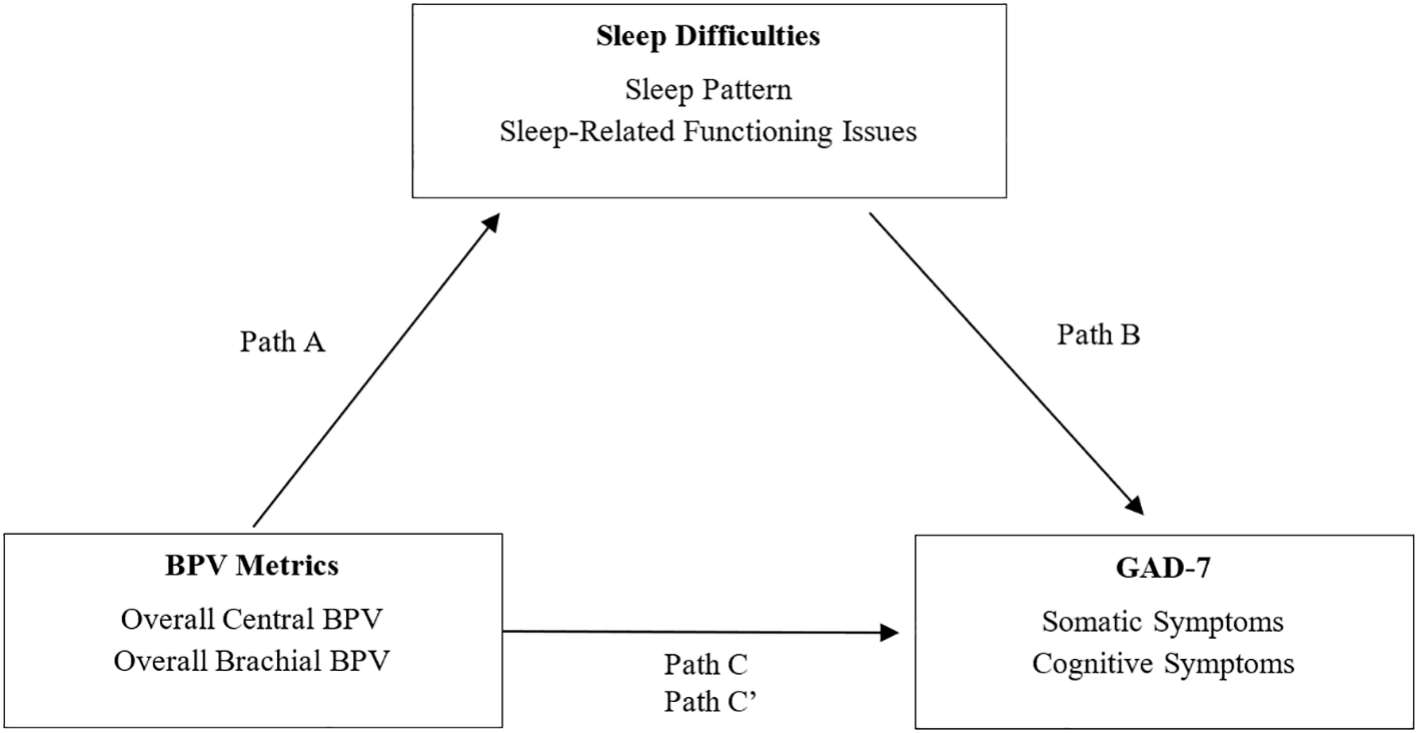
Mediation model of sleep difficulties on the relationship between BPV and GAD-7 symptoms. Mediation model illustrating the proposed role of sleep difficulties in the relationship between blood pressure variability (BPV) and anxiety symptoms (GAD-7). Path A reflects the association between BPV and sleep; Path B reflects the link between sleep and anxiety symptoms; Path C is the total effect, and Path C′ the indirect effect. Analyses are adjusted for age and gender.

**Figure 5** illustrates the mediating role of sleep quality (sleep patterns and sleep-related issues) in the BPV-GAD-7 symptom relationship, with adjustments for age and gender. Sleep-related issues emerged as a stronger mediator for both somatic and cognitive GAD-7 symptoms. Among central BPV metrics, AP and AIx were linked to better sleep-related functioning, which mediated their association with lower anxiety symptoms. AIx showed significant indirect effects on somatic (β = -0.01, p < 0.05) and cognitive (β = -0.02, p < 0.05) symptoms, while AP exhibited similar effects, particularly for cognitive symptoms (β = -0.02, p < 0.05). Conversely, brachial BPV metrics (MAP, diastolic BPV) were linked to worse sleep-related functioning, with brachial MAP showing significant indirect effects on cognitive symptoms (β = 0.06, p < 0.05), but no mediation effects for somatic symptoms. Sleep pattern was significantly associated with central PP for both GAD-7 symptom types, with higher PP linked to worse sleep patterns (**Tables 5**, **6**). However, no significant indirect effects were observed, suggesting sleep patterns play a weaker mediating role compared to sleep-related functioning issues.

**Table 5:** Mediation analysis of sleep quality, BPV and 24-hour somatic GAD-7 symptoms.

		Path A		Path B		Path C		Path C’	
		β	95% CI	β	95% CI	β	95% CI	β	95% CI
Sleep Pattern									
**Central**	SBP	0.09	(-0.39 - 0.57)	0.07*	(0.01 - 0.12)	-0.14*	(-0.26 - -0.02)	0.01	(-0.03 - 0.04)
	DBP	0.10	(-0.21 - 0.41)	0.07*	(0.01 - 0.12)	-0.07	(-0.15 - 0.01)	0.01	(-0.02 - 0.03)
	AP	0.03	(-0.04 - 0.10)	0.07**	(0.02 - 0.13)	-0.02*	(-0.04 - 0.00)	0.00	(0.00 - 0.01)
	MAP	-0.02	(-0.39 - 0.36)	0.06*	(0.01 - 0.12)	-0.12*	(-0.21 - -0.02)	0.00	(-0.03 - 0.03)
	PP	0.37*	(0.06 - 0.67)	0.07*	(0.01 - 0.12)	0.00	(-0.08 - 0.08)	0.02*	(0.00 - 0.06)
	AIx	0.01	(-0.06 - 0.08)	0.07*	(0.01 - 0.12)	-0.02*	(-0.04 - 0.00)	0.00	(0.00 - 0.01)
**Brachial**	SYS	0.20	(-0.25 - 0.66)	0.07*	(0.01 - 0.12)	-0.08	(-0.20 - 0.04)	0.01	(-0.02 - 0.05)
	DIA	0.17	(-0.07 - 0.41)	0.07*	(0.01 - 0.13)	-0.04	(-0.10 - 0.03)	0.01	(-0.01 - 0.03)
	MAP	0.22	(-0.12 - 0.56)	0.07*	(0.02 - 0.13)	-0.07	(-0.16 - 0.02)	0.02	(-0.01 - 0.04)
	PP	0.26	(-0.06 - 0.57)	0.07*	(0.01 - 0.12)	-0.01	(-0.09 - 0.07)	0.02	(0.00 - 0.05)
Sleep-Related Functioning Issues									
**Central**	SBP	0.05	(-0.13 - 0.22)	0.35**	(0.22 - 0.48)	-0.15**	(-0.26 - -0.05)	0.02	(-0.04 - 0.07)
	DBP	0.07	(-0.04 - 0.18)	0.36**	(0.23 - 0.49)	-0.09*	(-0.16 - -0.02)	0.02	(-0.01 - 0.07)
	AP	-0.03*	(-0.05 - 0.00)	0.32**	(0.18 - 0.46)	-0.01	(-0.03 - 0.01)	-0.01*	(-0.02 - 0.00)
	MAP	0.01	(-0.13 - 0.15)	0.34**	(0.21 - 0.47)	-0.12**	(-0.21 - -0.04)	0.00	(-0.04 - 0.05)
	PP	0.04	(-0.08 - 0.16)	0.34**	(0.20 - 0.47)	0.01	(-0.06 - 0.08)	0.01	(-0.03 - 0.06)
	AIx	-0.04**	(-0.06 - -0.01)	0.32**	(0.18 - 0.46)	-0.01	(-0.03 - 0.01)	-0.01*	(-0.02 - 0.00)
**Brachial**	SYS	0.12	(-0.05 - 0.29)	0.36**	(0.23 - 0.49)	-0.11*	(-0.22 - -0.01)	0.04	(-0.01 - 0.12)
	DIA	0.11*	(0.02 - 0.20)	0.38**	(0.24 - 0.52)	-0.07*	(-0.12 - -0.01)	0.04*	(0.01 - 0.09)
	MAP	0.14*	(0.01 - 0.26)	0.38**	(0.25 - 0.51)	-0.11**	(-0.19 - -0.03)	0.05*	(0.00 - 0.12)
	PP	0.05	(-0.06 - 0.17)	0.34**	(0.20 - 0.48)	-0.01	(-0.09 - 0.06)	0.02	(-0.02 - 0.07)

Caption; N = 88. Alx, Augmentation Index of Arterial Stiffness; Alx75, Augmentation Index of Arterial Stiffness normalised to heart rate of 75; AP, Augmentation Pressure; β, Mediation Coefficient; DBP, Diastolic Blood Pressure; GAD-7, Generalized Anxiety Disorder-7; MAP, Mean Arterial Pressure; PP Pulse Pressure, SBP Systolic Blood Pressure

Path A: BPV Metric to Sleep Quality (mediator) Path B: Sleep Quality (mediator) to Somatic GAD-7 Symptoms Path C: Total Effect Path C’: Direct Effect

Somatic Subscale: GAD-7 Items 4: Difficulty relaxing, Item 5: Restlessness, Item 6: Irritability.

Bolded values denoted *P < 0.05 ** P< 0.01.

**Table 6:** Mediation analysis of sleep quality factors, BPV and 24-hour cognitive GAD-7 symptoms.

		Path A		Path B		Path C		Path C’	
		β	95% CI	β	95% CI	β	95% CI	β	95% CI
Sleep Pattern									
**Central**	SBP	0.09	(-0.39 - 0.57)	0.11*	(0.02 - 0.19)	-0.10	(-0.28 - 0.09)	0.01	(-0.05 - 0.06)
	DBP	0.10	(-0.21 - 0.41)	0.11*	(0.02 - 0.19)	-0.01	(-0.13 - 0.11)	0.01	(-0.03 - 0.05)
	AP	0.03	(-0.04 - 0.10)	0.12**	(0.04 - 0.20)	-0.04**	(-0.06 - -0.01)	0.00	(0.00 - 0.01)
	MAP	-0.02	(-0.39 - 0.36)	0.11*	(0.02 - 0.19)	-0.05	(-0.20 - 0.10)	0.00	(-0.05 - 0.04)
	PP	0.37*	(0.06 - 0.67)	0.11*	(0.02 - 0.20)	-0.01	(-0.14 - 0.11)	0.04*	(0.00 - 0.09)
	AIx	0.01	(-0.06 - 0.08)	0.11**	(0.03 - 0.19)	-0.03*	(-0.06 - 0.00)	0.00	(-0.01 - 0.01)
**Brachial**	SYS	-0.02	(-0.07 - 0.04)	0.10*	(0.02 - 0.18)	-0.02*	(-0.04 - 0.00)	0.00	(-0.01 - 0.00)
	DIA	0.20	(-0.25 - 0.66)	0.11*	(0.02 - 0.19)	-0.02	(-0.20 - 0.16)	0.02	(-0.03 - 0.07)
	MAP	0.17	(-0.07 - 0.41)	0.11*	(0.02 - 0.19)	-0.02	(-0.12 - 0.08)	0.02	(-0.01 - 0.04)
	PP	0.22	(-0.12 - 0.56)	0.11*	(0.02 - 0.19)	-0.03	(-0.16 - 0.11)	0.02	(-0.01 - 0.06)
Sleep-Related Functioning Issues									
**Central**	SBP	0.05	(-0.13 - 0.22)	0.53**	(0.32 - 0.73)	-0.11	(-0.28 - 0.06)	0.02	(-0.06 - 0.11)
	DBP	0.07	(-0.04 - 0.18)	0.53**	(0.32 - 0.74)	-0.04	(-0.15 - 0.07)	0.04	(-0.02 - 0.10)
	AP	-0.03*	(-0.05 - 0.00)	0.48**	(0.27 - 0.69)	-0.02	(-0.05 - 0.00)	-0.01*	(-0.03 - 0.00)
	MAP	0.01	(-0.13 - 0.15)	0.52**	(0.31 - 0.73)	-0.06	(-0.19 - 0.08)	0.01	(-0.06 - 0.07)
	PP	0.04	(-0.08 - 0.16)	0.52**	(0.31 - 0.73)	0.00	(-0.11 - 0.12)	0.02	(-0.04 - 0.09)
	AIx	-0.04**	(-0.06 - -0.01)	0.49**	(0.28 - 0.71)	-0.01	(-0.04 - 0.01)	-0.02*	(-0.03 - 0.01)
**Brachial**	SYS	-0.03**	(-0.05 - -0.01)	0.49**	(0.27 - 0.70)	-0.01	(-0.03 - 0.01)	-0.01*	(-0.03 - 0.01)
	DIA	0.12	(-0.05 - 0.29)	0.53**	(0.32 - 0.74)	-0.06	(-0.23 - 0.10)	0.07	(-0.01 - 0.16)
	MAP	0.11*	(0.02 - 0.20)	0.56**	(0.35 - 0.77)	-0.06	(-0.15 - 0.03)	0.06*	(0.01 - 0.11)
	PP	0.14*	(0.01 - 0.26)	0.55**	(0.34 - 0.76)	-0.08	(-0.20 - 0.04)	0.08*	(0.01 - 0.15)

Caption N = 88. Alx Augmentation Index of Arterial Stiffness; Alx75 Augmentation Index of Arterial Stiffness normalised to heart rate of 75; AP Augmentation Pressure; β, Mediation Coefficient; BPV, Blood Pressure variability calculated using the coefficient of variation; DBP, Diastolic Blood Pressure; GAD-7, Generalized Anxiety Disorder-7; MAP, Mean Arterial Pressure; PP, Pulse Pressure; SBP, Systolic Blood Pressure;

Path A: BPV Metric to Sleep Quality (mediator) Path B: Sleep Quality (mediator) to Cognitive GAD-7 Symptoms Path C: Total Effect Path C’: Direct Effect.

Cognitive Subscale: GAD item 1: Nervousness, Item 2: Uncontrollable worry, Item 3: Excessive worry, Item 7: Feeling afraid.

Bolded values denoted *P < 0.05 ** P< 0.01.

## 4 Discussion

This study showed that central BPV showed stronger and more consistent associations with both cognitive and somatic anxiety symptoms, as well as total anxiety scores. Regression analysis confirmed these links, particularly with SBPV, MAP, and AIx across awake and overall periods. Brachial BPV showed no significant associations with anxiety symptoms. By contrast, BPV-depression associations were weaker and time-dependent, with no significant links to total depression scores. Central BPV correlated with depressive symptoms only during specific time periods, but regression analysis found no strong BPV-depression relationships, leading to the exclusion of PHQ-9 from mediation analysis. Higher BPV was linked to poorer sleep quality, with central and brachial PP as strong predictors of sleep disruptions. Sleep-related issues, not sleep patterns, mediated the BPV-anxiety relationship, particularly during the awake period. Brachial BPV had stronger links to sleep dysfunction, reinforcing its role in sleep-related distress.

### 4.1 BPV and anxiety

This study supports evidence that central BPV is associated with both cognitive and somatic anxiety symptoms, suggesting a common underlying mechanism involving autonomic and cerebrovascular dysfunction. While ANS disruptions are traditionally linked to somatic symptoms such as breathlessness and restlessness (11) and CBF instability to cognitive symptoms like excessive worry and impaired concentration (12), the similar BPV associations across symptom dimensions challenge this distinction. Instead, these findings suggest that BPV-related autonomic and cerebrovascular dysregulation may contribute broadly to anxiety symptoms rather than selectively to somatic or cognitive domains.

However, these findings contrast with most prior studies, which have reported positive associations between BPV and anxiety and have attributed this relationship to autonomic dysregulation, increased sympathetic activity, and reduced heart rate variability (31, 44–47). One potential explanation for these discrepancies is the variation in BPV measurement methods across studies. Many previous investigations have focused on brachial BPV, which may not fully capture cerebrovascular and autonomic contributions to anxiety. The current study’s inclusion of both central and brachial BPV offers a more comprehensive perspective due to its proximity to vital organs (22), reinforcing the importance of central BPV as a more physiologically relevant marker (20, 21). Differences in BPV measurement timeframes may also contribute to inconsistencies. Zhou et al. (2021) examined visit-to-visit BPV over a three-year period (47), whereas Virtanen et al. (2003) measured beat-to-beat BPV over just five minutes (44), neither of which accounted for nocturnal BPV or fluctuations over a 24-hour ambulatory period. The absence of circadian BP regulation in some studies may overlook key autonomic and cerebrovascular changes that influence anxiety symptoms (18, 25).

Longitudinal research further complicates the BPV-anxiety relationship. While acute anxiety is associated with increased BPV, likely due to baroreflex impairment, chronic anxiety has been linked to lower systolic BP over time, potentially leading to attenuated BPV (44, 48). This distinction underscores the importance of differentiating between acute and chronic anxiety in BPV research. Overall, these findings suggest that BPV-anxiety relationships depend on BPV type and symptom specificity. While brachial BPV and global anxiety scores have been linked to higher BPV, our results suggest that central BPV’s AIx, may reflect distinct autonomic patterns related to anxiety symptomatology. The difference in findings between central and brachial BPV may reflect underlying physiological differences. Central BPV, influenced by large artery stiffness and wave reflections, is more directly tied to cerebral perfusion and baroreflex sensitivity; key mechanisms implicated in emotional regulation and anxiety (44). In contrast, brachial BPV reflects peripheral pressure fluctuations that may be dampened or distorted due to arterial branching and muscular resistance (20). These differences underscore the importance of considering arterial measurement site when interpreting BPV-related associations, particularly for symptoms modulated by central autonomic networks.

### 4.2 BPV and depression

Unlike anxiety, BPV-depression associations were inconsistent, with no significant links to cognitive or somatic symptom dimensions. This finding does not align with research suggesting that BPV influences mood through autonomic and cerebrovascular mechanisms, particularly given the ANS’s role in physiological regulation and CBF’s involvement in cognitive and emotional processing (11, 12). However, when examining individual symptoms, hopelessness and psychomotor agitation were negatively associated with central BPV. This aligns with prior research indicating that hopelessness, independent of total depressive scores, is associated with BPV and vascular dysfunction (29). These findings suggest that examining individual depressive symptoms may provide greater insight into BPV-depression associations than broader cognitive and somatic dimensions.

Prior research remains inconsistent on the association between BPV with depression symptoms. Some studies propose that higher BPV contributes to depressive symptoms via cerebral microvascular dysfunction, autonomic instability, and cerebrovascular dysregulation (49, 50). However, others found no significant BPV differences between depressed and non-depressed individuals, suggesting a weaker or context-dependent BPV-depression link (44, 51). Differences in BPV measurement methods may partly explain the lack of consistent findings in previous research. Long-term BPV, particularly visit-to-visit BPV, reflects chronic vascular instability and has been more strongly linked to depression than short-term (24-hour) BPV (50). This suggests that prolonged exposure to high BPV, rather than short-term fluctuations, may contribute to depressive symptoms through mechanisms implicated in the vascular-depression hypothesis, which associates late-onset depression with cerebrovascular dysfunction and cognitive decline (50).

Demographic and lifestyle factors further complicate BPV-depression associations. Huang et al. (2020) found that higher systolic BPV was linked to depressive symptoms only after adjusting for BMI, smoking, and alcohol use (52), while Vallee et al. (2020) reported that lower BP and hypertension correlated with depression, with education level acting as a key modifying factor in women (53). These findings emphasize the need to control for individual differences when examining BPV in depression. While this study found limited evidence linking BPV to depression, the observed item-level associations suggest that future research should prioritize individual symptom analysis and long-term BPV measures comprehensively understand BPV’s role in depressive symptomatology.

### 4.3 BPV and sleep

Our findings indicate that BPV is associated with sleep disturbances, particularly sleep-related functioning rather than overall sleep quality. Lower central BPV metrics (SBP, MAP, and AIx) were linked to worse sleep-related functioning, while higher PP was associated with greater sleep disturbances, including frequent awakenings and poor sleep efficiency. These findings highlight distinct BPV metrics influencing different aspects of sleep dysfunction, reinforcing the need for granular BPV analysis. The association between higher PP and greater sleep disturbances aligns with evidence that arterial stiffness and wave reflection contribute to autonomic instability during sleep (54, 55). Similarly, sleep apnea, characterized by nocturnal BP oscillations, has been shown to amplify BPV, linking disrupted sleep architecture to heightened cardiovascular reactivity (56). Conversely, the relationship between lower BPV and increased sleep dysfunction may reflect reduced physiological flexibility in nocturnal BP regulation, as seen in individuals with insomnia and sleep apnea (57, 58).

The partial mediation of BPV-anxiety associations by sleep-related issues suggests that poor sleep functioning amplifies BPV-related anxiety symptoms, consistent with findings that sleep disturbances heighten sympathetic activation and cardiovascular reactivity (59). Mediation effects observed for AP and AIx indicate that arterial wave reflection may be a key hemodynamic mechanism linking sleep dysfunction to anxiety.

These findings align with evidence that poor sleep quality contributes to increased BPV (13) and that mood states influence nocturnal BP regulation, with negative moods (e.g., anger, sadness) linked to higher night time BP (60). Additionally, irregular sleep patterns have been associated with increased BP and hypertension risk, reinforcing the role of consistent sleep in cardiovascular stability (61). The absence of a mediation effect for depression supports the hypothesis that BPV is more relevant to autonomic regulation in anxiety than depression. Future research should investigate whether altered nighttime BP patterns contribute to long-term anxiety symptom severity and cardiovascular risk. These findings also have relevance for public health and clinical care. Central BPV may serve as a non-invasive marker of psychological distress in cardiovascular populations and inform early screening strategies. Moreover, interventions targeting sleep and autonomic regulation—such as Cognitive Behavioural Therapy for Insomnia (CBT-I) physical activity, and stress reduction—may help reduce BPV and anxiety symptoms, improving outcomes on both physiological and psychological fronts (54).

### 4.4 Strengths and limitations

This study presents several methodological strengths including the use of central BPV, which may provide a more accurate representation of cerebrovascular regulation and autonomic function compared to brachial BPV (20, 23). By incorporating multiple BPV indices over a 24-hour period, including MAP, PP and AIx, this study offers a comprehensive evaluation of short-term BP fluctuations in relation to depression, anxiety and sleep, and any possible physiological processes (62). Furthermore, 24-hour ABPM was utilized, which is superior to clinic-based or single-timepoint BP assessments, as it reduces white coat effects and captures real-world BP fluctuations that are more reflective of autonomic regulation (63). Another strength of this study is its symptom-specific analysis, differentiating between somatic and cognitive symptoms. Prior research has often generalized mood disturbances, which may obscure symptom-specific associations with BPV (29, 64).

A key limitation of this study is that analyses were largely exploratory in nature, and therefore no corrections for multiple comparisons were applied. While this may increase the risk of false positives, sensitivity in detecting potential associations was prioritised to inform hypotheses for future research (42, 43). Analyses were also constrained to using the CV, which does not capture the temporal association between BP measurements. In contrast, the average real variability (ARV) method takes into consideration the interrelated nature of successive measurements and is also associated with cognitive decline and cardiovascular risk (63, 65). Another limitation when using a 24-hour ambulatory BP monitoring to measure nocturnal BPV in a study where sleep was also quantified, is that the monitor itself may contribute to disrupted sleep and poorer sleep quality whilst simultaneously contributing to variance in nocturnal BP readings. Frequent disturbances from BP monitoring can lead to artificial fluctuations in nighttime BP measures, potentially influencing sleep-dependent autonomic regulation (57).

This study also did not standardize physical activity or dietary intake, both of which influence BPV, mood, and sleep. Caffeine intake, in particular, has been shown to increase BP fluctuations, potentially affecting the cardiovascular system’s regulatory mechanisms (66). Additionally, consuming 200–300 mg of caffeine has been shown to increase systolic BP by approximately 8.1 mmHg and diastolic BP by 5.7 mmHg, with effects lasting at least three hours (67, 68). The lack of control over dietary intake, including caffeine consumption, may have introduced unaccounted variability in BPV findings. Similarly, while hypertension medication use was recorded, it was not statistically adjusted for. Antihypertensive medications affect vascular tone, autonomic responses, and BPV, potentially influencing BPV-mood relationships (69). Future research should incorporate a more comprehensive set of covariates to account for confounding factors.

Additionally, the study was constrained by a small sample size, reducing statistical power and increasing the risk of Type II errors (70). The broad age range (23–86 years, median 57.6 years) may also dilute age-specific effects, particularly as BPV, mood, and cognitive function are known to vary across the lifespan (47). While age was included as a covariate in adjusted models, age-stratified analyses may provide greater specificity in detecting BPV-mood interactions. Gender differences were also not explicitly analyzed, and it has been shown that BPV-mood relationships may differ between males and females due to hormonal and vascular factors (47). Vallee et al. (2020) reported that lower BP and hypertension correlated with depression, with education level acting as a key modifying factor in women (53, 71). These findings emphasize the importance of controlling for demographic and lifestyle factors when examining BPV-psychological health interactions. These limitations may affect the strength and generalizability of our conclusions. Future studies should use longitudinal designs to clarify temporal relationships, apply age- and gender-stratified analyses, and control for lifestyle factors such as caffeine, physical activity, and medication adherence to reduce confounding and enhance causal interpretation (63, 69).

Finally, the cross-sectional design significantly limits causal inferences. This is particularly relevant to the mediation analyses, which assume a temporal sequence between variables that cannot be confirmed within a cross-sectional framework. Longitudinal research is needed to clarify whether BPV changes precede or follow mood disturbances and to establish bidirectional relationships between BPV, sleep, and psychological health (72, 73). Future studies should also integrate alternative BPV metrics (e.g., ARV and HRV) to enhance measurement precision. Interventional research targeting BPV modulation—via exercise, dietary changes, and stress reduction techniques—could further clarify the association between BPV and psychological distress. Expanding statistical models through propensity score matching and structural equation modelling will improve control for unmeasured confounders (e.g. education level, BMI, caffeine intake and antihypertensive use). Additionally, age- and gender-stratified analyses and recruitment from diverse community-based populations will improve generalizability. By addressing these gaps, future research can refine BPV’s role as a dynamic marker of psychological and autonomic regulation and explore its potential as a therapeutic target for mood and sleep disturbances.

### 4.5 Conclusion

This study indicates that central BPV showed stronger and more consistent associations with anxiety symptoms than depression, reinforcing its relevance in autonomic and cerebrovascular dysregulation. In contrast, BPV-depression associations were weak and inconsistent, suggesting that BPV may be more relevant to individual depressive symptoms, such as hopelessness and psychomotor agitation, rather than broader symptom dimensions. Additionally, sleep disturbances, particularly sleep-related functioning, mediated the BPV-anxiety relationship, highlighting the role of sleep in autonomic regulation and psychological distress. By incorporating central BPV metrics and circadian BPV patterns, this study advances understanding of the physiological mechanisms linking BPV to mental health. Future research should employ longitudinal designs to clarify causal pathways, explore alternative BPV metrics such as visit-to-visit variability, and examine sex- and age-specific effects. Investigating additional confounders, including medication and lifestyle factors, may further refine BPV’s role in psychological distress. Given the mediating role of sleep, future research should investigate whether interventions targeting sleep quality and autonomic regulation could benefit both mental and cardiovascular health.

## Data Availability

The raw data supporting the conclusions of this article will be made available by the authors, without undue reservation.
