# Supplementary material for: Beyond the beats: exploring the link between blood pressure fluctuations with anxiety, depression, and sleep
Source: Front Psychiatry. 2025 Jul 31;16:1595979. doi: 10.3389/fpsyt.2025.1595979 (PMC12352328; doi:10.3389/fpsyt.2025.1595979)
Supplement: Supplementary file 1 [file Table1.docx]

Supplementary Material

| **Supplementary Table 1.**  *Spearman’s Correlation of BPV and Anxiety Scores* | | | | | | | | | | |
| --- | --- | --- | --- | --- | --- | --- | --- | --- | --- | --- |
|  | |  | **GAD-7 Items** | | | | | | | |
|  |  |  | **Q1** | **Q2** | **Q3** | **Q4** | **Q5** | **Q6** | **Q7** | **Total** |
| **Time** | **BPV Phenotypes** | | ρ | ρ | ρ | ρ | ρ | ρ | ρ | ρ |
| **Overall** | **Central** | SBP | **-0.21*** | -0.15 | -0.08 | **-0.28**** | -0.18 | -0.17 | **-0.22*** | **-0.27*** |
|  |  | DBP | -0.04 | 0.10 | 0.08 | -0.03 | -0.13 | -0.17 | -0.03 | -0.06 |
|  |  | AP | -0.09 | -0.15 | -0.14 | -0.03 | 0.08 | **-0.22*** | -0.16 | -0.15 |
|  |  | MAP | -0.09 | -0.02 | 0.01 | -0.11 | -0.14 | **-0.25*** | -0.10 | -0.16 |
|  |  | PP | -0.08 | -0.04 | -0.02 | -0.01 | -0.02 | 0.02 | 0.05 | -0.06 |
|  |  | AIx | 0.03 | -0.04 | -0.07 | 0.07 | 0.15 | -0.12 | -0.06 | -0.02 |
|  | **Brachial** | SYS | -0.06 | 0.00 | 0.00 | -0.16 | -0.07 | -0.02 | -0.17 | -0.12 |
|  |  | DIA | -0.04 | 0.16 | 0.02 | -0.04 | -0.10 | -0.15 | -0.04 | -0.06 |
|  |  | MAP | -0.05 | 0.10 | 0.00 | -0.11 | -0.09 | -0.10 | -0.12 | -0.08 |
|  |  | PP | -0.13 | -0.10 | -0.02 | -0.09 | -0.13 | 0.03 | -0.08 | -0.14 |
| **Awake** | **Central** | SBP | -0.02 | -0.17 | -0.10 | -0.09 | 0.03 | 0.05 | -0.03 | -0.08 |
|  |  | DBP | 0.01 | 0.06 | 0.05 | 0.03 | -0.07 | -0.09 | 0.05 | 0.01 |
|  |  | AP | -0.04 | -0.09 | -0.13 | 0.02 | 0.16 | -0.15 | -0.08 | -0.08 |
|  |  | MAP | 0.00 | -0.07 | -0.03 | -0.02 | -0.03 | -0.05 | 0.00 | -0.05 |
|  |  | PP | -0.14 | -0.08 | -0.13 | -0.10 | -0.01 | 0.04 | 0.00 | -0.10 |
|  |  | AIx | 0.02 | -0.01 | -0.07 | 0.06 | 0.19 | -0.16 | -0.04 | -0.04 |
|  | **Brachial** | SYS | 0.04 | -0.06 | -0.01 | 0.00 | 0.08 | 0.16 | 0.03 | 0.01 |
|  |  | DIA | 0.00 | 0.08 | 0.01 | 0.03 | -0.02 | -0.05 | 0.09 | 0.00 |
|  |  | MAP | 0.04 | 0.04 | 0.02 | 0.03 | -0.01 | 0.02 | 0.05 | 0.01 |
|  |  | PP | -0.16 | -0.07 | -0.06 | -0.08 | -0.11 | 0.05 | -0.07 | -0.13 |
| **Asleep** | **Central** | SBP | 0.01 | 0.00 | 0.07 | -0.07 | -0.11 | -0.06 | 0.06 | -0.04 |
|  |  | DBP | 0.06 | 0.04 | 0.06 | 0.01 | -0.05 | -0.02 | 0.03 | 0.00 |
|  |  | AP | -0.05 | -0.04 | -0.01 | 0.03 | 0.05 | -0.09 | -0.08 | -0.02 |
|  |  | MAP | 0.03 | 0.01 | 0.07 | -0.03 | -0.07 | -0.09 | 0.05 | -0.06 |
|  |  | PP | 0.01 | 0.03 | 0.10 | 0.10 | 0.04 | -0.03 | 0.13 | 0.06 |
|  |  | AIx | -0.10 | -0.09 | -0.04 | -0.01 | 0.01 | -0.13 | -0.16 | -0.10 |
|  | **Brachial** | SYS | 0.05 | 0.05 | 0.11 | -0.02 | -0.06 | 0.02 | 0.02 | 0.03 |
|  |  | DIA | 0.11 | 0.15 | 0.08 | 0.02 | -0.04 | -0.05 | 0.00 | 0.06 |
|  |  | MAP | 0.08 | 0.15 | 0.12 | -0.02 | -0.07 | -0.07 | -0.03 | 0.04 |
|  |  | PP | 0.01 | 0.03 | 0.12 | 0.11 | 0.02 | 0.00 | 0.09 | 0.04 |
| *Caption. N = 88. Alx, Augmentation Index of Arterial Stiffness; AP, Augmentation Pressure; DBP, Diastolic Blood Pressure; GAD-7, Generalized Anxiety Disorder-7; MAP, Mean Arterial Pressure; PP, Pulse Pressure; ρ, Spearman’s rho correlation coefficient; SBP, Systolic Blood Pressure*  *Bolded values * P < 0.05 ** P < 0.01*  *Q1. Feeling nervous, anxious, or on edge*  *Q2. Not being able to stop or control worrying*  *Q3. Worrying too much about different things*  *Q4. Trouble relaxing*  *Q5. Being so restless that it is hard to sit still*  *Q6. Becoming easily annoyed or irritable*  *Q7. Feeling afraid, as if something awful might happen* | | | | | | | | | | |

| **Supplementary Table 2.**  *Spearman’s Correlation of BPV and Depression Scores* | | | | | | | | | | | | |
| --- | --- | --- | --- | --- | --- | --- | --- | --- | --- | --- | --- | --- |
|  |  |  | **PHQ-9 Items** | | | | | | | | | |
|  |  |  | **Q1** | **Q2** | **Q3** | **Q4** | **Q5** | **Q6** | **Q7** | **Q8** | **Q9** | **Total** |
| **Time** | **BPV Phenotypes** | | ρ | ρ | ρ | ρ | ρ | ρ | ρ | ρ | ρ | ρ |
| **Overall** | **Central** | SBP | -0.01 | **-0.23*** | 0.00 | 0.18 | -0.12 | -0.08 | 0.12 | **-0.22*** | -0.03 | -0.02 |
|  |  | DBP | 0.00 | -0.16 | -0.04 | 0.06 | 0.03 | -0.05 | 0.03 | -0.15 | -0.03 | -0.04 |
|  |  | AP | -0.09 | -0.03 | -0.01 | -0.01 | -0.11 | -0.04 | -0.03 | 0.11 | -0.07 | -0.07 |
|  |  | MAP | 0.04 | -0.20 | -0.01 | 0.12 | -0.01 | -0.02 | 0.06 | **-0.21*** | 0.02 | -0.02 |
|  |  | PP | -0.19 | 0.02 | 0.03 | 0.00 | 0.01 | -0.01 | -0.02 | 0.00 | -0.17 | 0.00 |
|  |  | AIx | -0.07 | -0.05 | -0.08 | 0.02 | -0.03 | -0.08 | -0.01 | 0.15 | -0.07 | -0.08 |
|  | **Brachial** | SYS | 0.09 | -0.20 | 0.03 | 0.18 | -0.07 | 0.00 | 0.09 | -0.08 | -0.04 | 0.06 |
|  |  | DIA | 0.07 | -0.10 | 0.01 | 0.07 | 0.08 | 0.04 | 0.11 | -0.04 | -0.07 | 0.06 |
|  |  | MAP | 0.11 | -0.16 | 0.03 | 0.13 | 0.08 | 0.01 | 0.11 | -0.06 | -0.06 | 0.07 |
|  |  | PP | -0.08 | -0.02 | -0.07 | 0.03 | -0.09 | 0.02 | -0.04 | -0.10 | -0.11 | -0.03 |
| **Awake** | **Central** | SBP | 0.08 | 0.05 | 0.08 | 0.18 | -0.12 | 0.02 | 0.08 | -0.07 | -0.09 | 0.11 |
|  |  | DBP | -0.04 | -0.02 | 0.01 | 0.07 | 0.00 | 0.02 | 0.05 | -0.08 | -0.11 | 0.03 |
|  |  | AP | 0.00 | 0.03 | -0.04 | -0.03 | -0.08 | -0.04 | -0.03 | 0.16 | -0.09 | -0.05 |
|  |  | MAP | 0.01 | 0.01 | 0.08 | 0.14 | -0.06 | 0.04 | 0.08 | -0.08 | -0.11 | 0.10 |
|  |  | PP | -0.17 | -0.01 | -0.04 | 0.01 | -0.08 | -0.10 | -0.05 | 0.00 | -0.17 | -0.07 |
|  |  | AIx | 0.05 | -0.03 | -0.10 | 0.18 | -0.02 | 0.01 | -0.05 | **0.23*** | -0.13 | -0.06 |
|  | **Brachial** | SYS | 0.10 | 0.05 | 0.09 | **0.24*** | -0.05 | 0.09 | 0.09 | 0.02 | -0.11 | 0.19 |
|  |  | DIA | 0.03 | 0.02 | 0.02 | 0.09 | 0.06 | 0.09 | 0.15 | 0.00 | -0.13 | 0.10 |
|  |  | MAP | 0.10 | 0.02 | 0.07 | 0.18 | 0.05 | 0.11 | 0.13 | 0.00 | -0.11 | 0.17 |
|  |  | PP | -0.08 | -0.02 | -0.13 | 0.02 | -0.11 | -0.01 | -0.08 | -0.09 | -0.14 | -0.08 |
| **Asleep** | **Central** | SBP | -0.08 | -0.15 | 0.13 | 0.08 | -0.10 | -0.13 | 0.10 | -0.12 | 0.13 | -0.02 |
|  |  | DBP | 0.03 | -0.15 | 0.02 | 0.07 | 0.05 | -0.08 | 0.08 | -0.10 | 0.20 | -0.02 |
|  |  | AP | -0.16 | **-0.27*** | 0.06 | 0.05 | **-0.21*** | -0.14 | -0.02 | 0.01 | -0.01 | -0.08 |
|  |  | MAP | 0.00 | -0.18 | 0.10 | 0.08 | 0.00 | -0.09 | 0.07 | -0.10 | 0.21 | -0.02 |
|  |  | PP | **-0.21*** | -0.08 | 0.12 | -0.03 | 0.02 | -0.08 | -0.05 | -0.04 | -0.12 | 0.03 |
|  |  | AIx | **-0.22*** | **-0.26*** | 0.08 | 0.08 | -0.13 | -0.17 | -0.13 | 0.00 | 0.00 | -0.09 |
|  | **Brachial** | SYS | -0.06 | -0.14 | 0.09 | 0.04 | -0.14 | -0.08 | 0.10 | -0.05 | 0.10 | 0.00 |
|  |  | DIA | 0.12 | -0.14 | 0.02 | 0.05 | 0.07 | -0.06 | 0.09 | -0.02 | 0.20 | 0.02 |
|  |  | MAP | 0.09 | -0.18 | 0.04 | 0.10 | -0.01 | -0.09 | 0.09 | -0.04 | 0.20 | 0.01 |
|  |  | PP | -0.14 | -0.05 | 0.07 | -0.03 | 0.04 | -0.02 | -0.10 | 0.00 | -0.21 | 0.04 |
| *Caption. N = 88. Alx, Augmentation Index of Arterial Stiffness; AP, Augmentation Pressure; DBP, Diastolic Blood Pressure; MAP, Mean Arterial Pressure; PHQ-9, Patient Health Questionnaire-9; PP, Pulse Pressure; ρ, Spearman’s rho correlation coefficient; SBP, Systolic Blood Pressure*  *Bolded values * P < 0.05 ** P < 0.01*  *PHQ-9 Items*  *Q1. Little interest or pleasure in doing things*  *Q2. Feeling down, depressed, or hopeless*  *Q3. Trouble falling or staying asleep, or sleeping too much*  *Q4. Feeling tired or having little energy*  *Q5. Poor appetite or overeating*  *Q6. Feeling bad about yourself or that you are a failure or have let yourself or your family down*  *Q7. Trouble concentrating on things, such as reading the newspaper or watching television*  *Q8. Moving or speaking so slowly that other people could have noticed. Or the opposite being so fidgety or restless that you have been moving around a lot more than usual*  *Q9. Thoughts that you would be better off dead, or of hurting yourself* | | | | | | | | | | | | |

| **Supplementary Table 3.**  *Spearman’s Correlation of BPV and Sleep Quality Reversed* | | | | | | | | | | | |
| --- | --- | --- | --- | --- | --- | --- | --- | --- | --- | --- | --- |
|  | |  | **SCI Items** | | | | | | | | |
|  |  |  | **Q1** | **Q2** | **Q3** | **Q4** | **Q5** | **Q6** | **Q7** | **Q8** | **Total** |
| **Time** | **BPV Phenotypes** | | ρ | ρ | ρ | ρ | ρ | ρ | ρ | ρ | ρ |
| **Overall** | **Central** | SBP | -0.01 | 0.00 | -0.04 | -0.01 | 0.00 | -0.02 | -0.04 | 0.03 | 0.00 |
|  |  | DBP | 0.06 | -0.06 | -0.01 | 0.17 | 0.15 | 0.14 | 0.10 | 0.12 | 0.11 |
|  |  | AP | -0.11 | 0.09 | 0.07 | 0.00 | -0.15 | -0.09 | -0.09 | 0.02 | 0.01 |
|  |  | MAP | -0.02 | -0.13 | -0.07 | 0.09 | 0.04 | 0.02 | -0.01 | 0.03 | -0.01 |
|  |  | PP | 0.00 | **0.23*** | 0.21 | **0.21*** | -0.04 | -0.02 | 0.06 | **0.25*** | 0.18 |
|  |  | AIx | -0.15 | -0.02 | 0.03 | -0.03 | -0.08 | -0.03 | -0.07 | 0.00 | -0.02 |
|  | **Brachial** | SYS | -0.02 | 0.10 | 0.01 | 0.04 | 0.13 | 0.05 | 0.11 | 0.07 | 0.09 |
|  |  | DIA | 0.10 | 0.08 | 0.02 | **0.23*** | 0.19 | 0.20 | 0.19 | 0.13 | 0.19 |
|  |  | MAP | 0.08 | 0.05 | 0.03 | 0.17 | 0.20 | 0.16 | 0.18 | 0.11 | 0.17 |
|  |  | PP | -0.08 | 0.16 | 0.09 | 0.14 | -0.04 | -0.06 | 0.02 | 0.11 | 0.07 |
| **Awake** | **Central** | SBP | 0.06 | 0.14 | 0.13 | 0.07 | 0.19 | 0.11 | 0.12 | 0.13 | 0.15 |
|  |  | DBP | 0.08 | -0.03 | 0.07 | 0.19 | **0.21*** | 0.17 | 0.18 | 0.18 | 0.17 |
|  |  | AP | -0.11 | 0.03 | 0.09 | 0.04 | -0.11 | -0.08 | -0.10 | 0.04 | 0.02 |
|  |  | MAP | 0.06 | -0.01 | 0.06 | 0.13 | 0.19 | 0.14 | 0.14 | 0.14 | 0.14 |
|  |  | PP | -0.05 | **0.21*** | 0.16 | 0.15 | -0.08 | -0.10 | -0.05 | 0.18 | 0.11 |
|  |  | AIx | -0.12 | -0.03 | 0.06 | 0.05 | -0.09 | -0.07 | -0.09 | -0.02 | 0.00 |
|  | **Brachial** | SYS | 0.01 | 0.16 | 0.12 | 0.11 | **0.25*** | 0.17 | 0.17 | 0.11 | 0.17 |
|  |  | DIA | 0.10 | 0.07 | 0.06 | 0.20 | **0.22*** | **0.22*** | **0.24*** | 0.15 | 0.20 |
|  |  | MAP | 0.09 | 0.12 | 0.08 | 0.19 | **0.24*** | **0.23*** | **0.23*** | 0.14 | **0.21*** |
|  |  | PP | -0.09 | 0.08 | 0.06 | 0.13 | 0.01 | -0.03 | -0.01 | 0.08 | 0.04 |
| **Asleep** | **Central** | SBP | 0.02 | 0.08 | 0.06 | 0.05 | 0.01 | 0.08 | 0.06 | 0.04 | 0.08 |
|  |  | DBP | -0.04 | 0.00 | 0.05 | 0.11 | 0.06 | 0.12 | 0.02 | 0.01 | 0.05 |
|  |  | AP | -0.01 | 0.09 | 0.12 | -0.03 | -0.07 | -0.01 | 0.03 | 0.00 | 0.05 |
|  |  | MAP | -0.05 | 0.02 | 0.03 | 0.04 | -0.01 | 0.08 | -0.01 | -0.02 | 0.01 |
|  |  | PP | 0.00 | 0.05 | 0.16 | 0.10 | 0.03 | 0.04 | 0.17 | 0.15 | 0.13 |
|  |  | AIx | -0.06 | 0.08 | 0.09 | 0.02 | -0.08 | -0.02 | -0.06 | 0.02 | 0.05 |
|  | **Brachial** | SYS | -0.03 | 0.03 | 0.09 | 0.06 | 0.10 | 0.11 | 0.18 | 0.07 | 0.11 |
|  |  | DIA | -0.05 | -0.04 | -0.01 | 0.12 | 0.14 | 0.13 | 0.07 | 0.02 | 0.08 |
|  |  | MAP | -0.02 | -0.03 | -0.02 | 0.04 | 0.07 | 0.09 | 0.06 | 0.01 | 0.06 |
|  |  | PP | 0.00 | 0.04 | 0.17 | 0.15 | -0.01 | 0.03 | 0.16 | 0.14 | 0.12 |
| *Caption. N = 88. Alx, Augmentation Index of Arterial Stiffness; AP, Augmentation Pressure; DBP, Diastolic Blood Pressure; MAP, Mean Arterial Pressure; PP, Pulse Pressure; ρ, Spearman’s rho correlation coefficient; SBP, Systolic Blood Pressure; SCI, Sleep Condition Indicator*  *Bolded values * P < 0.05 ** P < 0.01*  *SCI items,*  *Q1. How long does it take you to fall asleep?*  *Q2. If you then wake up during the night ... how long are you awake for in total? (add up all the awakenings)*  *Q3. How many nights a week do you have a problem with your sleep?*  *Q4. How would you rate your sleep quality?*  *Q5. Thinking about the past month, to what extent has poor sleep: Affected your mood, energy, or relationships?*  *Q6. Affected your concentration, productivity, or ability to stay awake?*  *Q7. Troubled you in general?*  *Q8. Finally: How long have you had a problem with your sleep?* | | | | | | | | | | | |
